# Supplementary material for: Whole-Genome Inter-Sex Variation in Russian Sturgeon (Acipenser gueldenstaedtii)
Source: Int J Mol Sci. 2022 Aug 22;23(16):9469. doi: 10.3390/ijms23169469 (PMC9409348; doi:10.3390/ijms23169469)
Supplement: Supplementary file 1 [file ijms-23-09469-s001.zip › Figure S1.pdf]

Figure S1

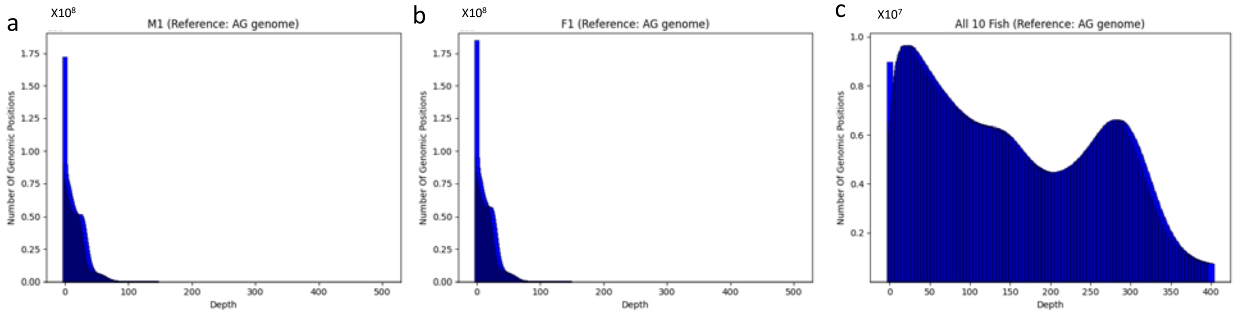

Figure S1: Distributions of sequencing depth over all genome positions when reads are aligned to the **AG genome**: (a) Male M1; (b) Female F1; (c) All samples combined.
